# Chest-MRI under pulsatile flow ventilation: A new promising technique

**DOI:** 10.1371/journal.pone.0178807

**Published:** 2017-06-12

**Authors:** Catherine Beigelman-Aubry, Nicolas Peguret, Matthias Stuber, Jean Delacoste, Bastien Belmondo, Alban Lovis, Julien Simons, Olivier Long, Kathleen Grant, Gregoire Berchier, Chantal Rohner, Gabriele Bonanno, Simone Coppo, Juerg Schwitter, Mahmut Ozsahin, Salah Qanadli, Reto Meuli, Jean Bourhis

**Affiliations:** 1 Department of Radiology, CHUV and University of Lausanne, Lausanne, Switzerland; 2 Department of Radiation Oncology, CHUV and University of Lausanne, Lausanne, Switzerland; 3 Center for biomedical Imaging (CIBM), Lausanne, Switzerland; 4 Department of Physiotherapy, CHUV and University of Lausanne, Lausanne, Switzerland; 5 Department of Pneumology, CHUV and University of Lausanne, Lausanne, Switzerland; 6 Division of Cardiology, CHUV and University of Lausanne, Lausanne, Switzerland; 7 Cardiac MR center, CHUV and University of Lausanne, Lausanne, Switzerland; Northwestern University Feinberg School of Medicine, UNITED STATES

## Abstract

**Objectives:**

Magnetic resonance imaging (MRI) of the chest has long suffered from its sensitivity to respiratory and cardiac motion with an intrinsically low signal to noise ratio and a limited spatial resolution. The purpose of this study was to perform chest MRI under an adapted non invasive pulsatile flow ventilation system (high frequency percussive ventilation, HFPV^®^) allowing breath hold durations 10 to 15 times longer than other existing systems.

**Methods:**

One volunteer and one patient known for a thymic lesion underwent a chest MRI under ventilation percussion technique (VP-MR). Routinely used sequences were performed with and without the device during three sets of apnoea on inspiration.

**Results:**

VP-MR was well tolerated in both cases. The mean duration of the thoracic stabilization was 10.5 min (range 8.5–12) and 5.8 min (range 5–6.2) for Volunteer 1 and Patient 1, respectively. An overall increased image quality was seen under VP-MR with a better delineation of the mediastinal lesion for Patient 1. Nodules discovered in Volunteer 1 were confirmed with low dose CT.

**Conclusion:**

VP-MR was feasible and increased spatial resolution of chest MRI by allowing acquisition at full inspiration during thoracic stabilization approaching prolonged apnoea. This new technique could be of benefit to numerous thoracic disorders.

## Introduction

Magnetic resonance imaging (MRI), a radiation-free technique, has long suffered from the low proton density and high magnetic susceptibility of the lungs [1]. These characteristics explain the intrinsically low signal to noise ratio and limited spatial resolution with a great sensitivity to respiratory and cardiac motions, that are most prominent in the lower and anterior sections of the chest [2]. Repetitive breath holds of around 20 seconds each are required for most MR acquisitions split into several blocks with possible additional artefacts in case of irreproducible breath holds [2]. Dedicated techniques for respiratory motion compensation have also been developed, including respiratory belts, navigator [3] and most recently 3D free-breathing isotropic radial sequences with motion compensation [4]. This unfortunately prolongs the acquisition time, with an imperfect compensation [2] and acquisitions usually performed at end of normal expiration [4]. Moreover, although sequences with parallel acquisition techniques have been developed, limitation in spatial resolution still remains, suggesting the need for developing novel approaches to suppress respiratory artefacts.

Of particular interest in this field is the method of high frequency percussive ventilation (HFPV^®^), developed by Forrest M. Bird, which is based on the administration of small volumes of air so called “percussions” with adjustable pressures and frequencies. These high-frequency percussions can be superimposed on (and replace) spontaneous ventilation. The system is pneumatic, works without electronics and is not influenced by electromagnetic forces. The security of the system is based on the fact that percussions are administered through an open circuit called Phasitron, composed of a sliding venturi which delivers percussive tidal volumes according to the current subject’s thoraco-pulmonary compliance [5–7]. At each high frequency insufflation the system delivers low volumes at high pressure when compliance is low and high volumes at low pressure when compliance is high. In case of increased pressure or reduced thoraco-pulmonary compliance, the piston moves back leaving the expiratory port open, enabling protection against overpressure. HFPV^®^ has been shown to improve oxygenation compared to conventional ventilation and has been widely used in Medicine for various acute and chronic situations in all age groups (from newborn to the elderly) [8,9]. A modified HFPV^®^ technique was recently tested in patients for whom chest immobilization was needed as part of their thoracic radiotherapy. This pilot clinical study was approved by our ethical committee (Protocol 225/14 CHUV-DO-PART) and showed that Percussion Assisted Radiotherapy was feasible, well tolerated, reproducible and potentially associated with dosimetric gains compared to other techniques for controlling lung motion. In a series of 50 consecutive radiotherapy fractions, prolonged apnoea-like lung stabilization could be obtained in 100% of the cases with a mean duration of chest stabilization of 8.3 minutes [10].

Based on this positive experience, it was decided to adapt this non invasive technique for chest MRI. The goal of our study was to evaluate the feasibility and potential benefit of combining this percussive ventilation with chest MRI, so called VP-MR, in order to assess the effect of prolonged thoracic stabilization at full inspiration on the quality of MR images.

## Methods and materials

The realization of the pilot study for testing HFPV^®^ was authorized by the Ethics Committee on Human Research of the State of Vaud (Protocol 225/14 CHUV-DO-PART). The technique was applied on one volunteer and one patient who gave their written informed consent to this study, in accordance with the ethics committee requirements.

### Device

**Fig 1:**
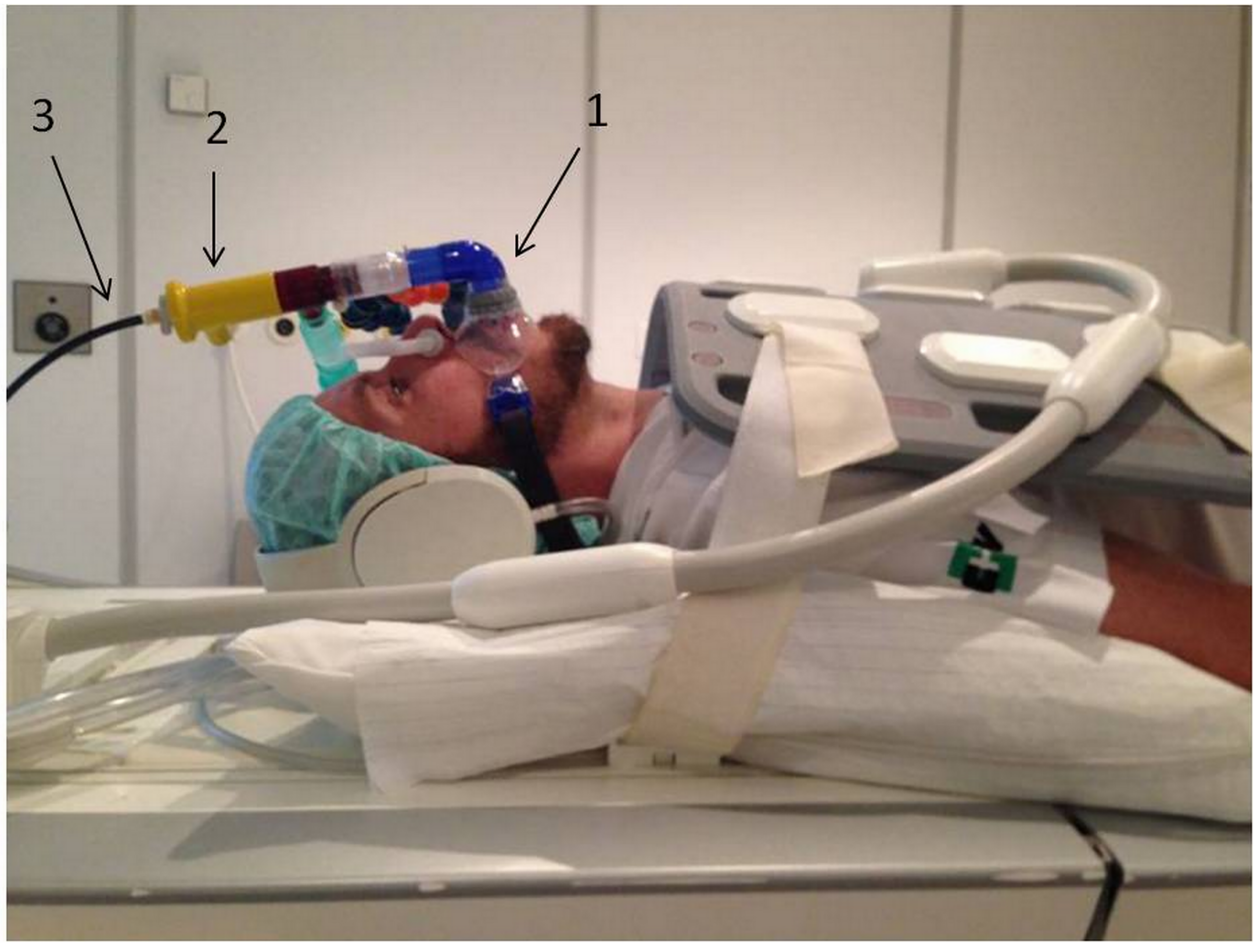
High frequency non invasive ventilation in an awake non-sedated subject lying on a MRI table. The Percussionaire^®^ ventilator remains outside the room and is connected to the non invasive interface (mouthpiece (1)) via the Phasitron (2) using a 6 meter long circuit (3).

The HFPV^®^ was obtained via a Transrespirator^®^, (Percussionaire^®^ Corporation; Idaho, USA). This Transrespirator^®^ was adapted to be MRI compatible, and one metallic component of the Phasitron was replaced by a MRI compatible part. Six meter long tubes were adapted for use with the HFPV^®^ allowing the installation of the Transpirator^®^ outside the MRI room (Fig 1). The settings were chosen as follows: airflow was set to obtain airway pressure between 20 and 40 cm H_2_O; frequency between 400 and 500 cycles per minute; FiO_2_ was set at 100%; work pressure was set between 20 and 40 PSI. The inspiratory and expiratory times were adjusted to eliminate the variations of the Pressure Controlled Ventilation (PCV) and continuous positive airway pressure (CPAP) demand was set at 0 cmH_2_O.

Conventional use of HFPV^®^ superimposes high frequency ventilation on a PCV type mode. In this study we eliminated the PCV component and ventilated the subject only by tiny variations of the pressure using high frequency setting in order to obtain thoracic stabilization approaching apnoea. Owing to the positive pressure used, the lungs were explored at inspiratory volumes.

### MRI feasibility

Volunteer 1, 38 y-old, was in good health with no previous known disorder. Patient 1, 55 y-old, had a history of a thymic lesion known to be stable at previous examinations and requiring CT or MR control. In order to avoid the use of ionizing radiation, a less invasive chest MRI was proposed instead of the CT-scan for the follow-up of the lesion. No contrast administration was used in either case. Both examinations were performed using Siemens 1.5T equipment (Aera, Siemens, Erlangen, Germany), at full inspiration and using cardiac gating in order to eliminate cardiac artefacts. The arms were positioned along the body. For each MRI exam, the same sequences were performed with and without the respiratory device (Table 1). Sequences with compensatory corrections of motion, e.g. BLADE were not used. The sequences in thoracic stabilization were split into three sets, while the overall time of MR exam was intended to be 45–60 minutes in both cases. Careful combinations of sequences were chosen in order to take optimal advantage of each stabilization period.

**Table 1:** MR Sequences performed under VP and without the device.

Volunteer 1		Patient 1	
Sequences	Slice thickness	Sequences	Slice thickness
Fast Turbo spin echo (Axial)	2 mm	T2 Turbo spin echo (Coronal)	3 mm
T1 Vibe (Axial)	1.50 mm	T1 Vibe TE: 0.8 sec (Axial, coronal)	4 mm
Free running ultra-short echo time	1.30 mm	Diffusion (b50-800)	6 mm
3D radial SSFP	1.15 mm	Free running ultra-short echo time	1.3 mm
Cine sequences (Axial)	4 mm	3D radial SSFP	1.3 mm
		TRUFI 3D	1.3mm

**Fig 2:**
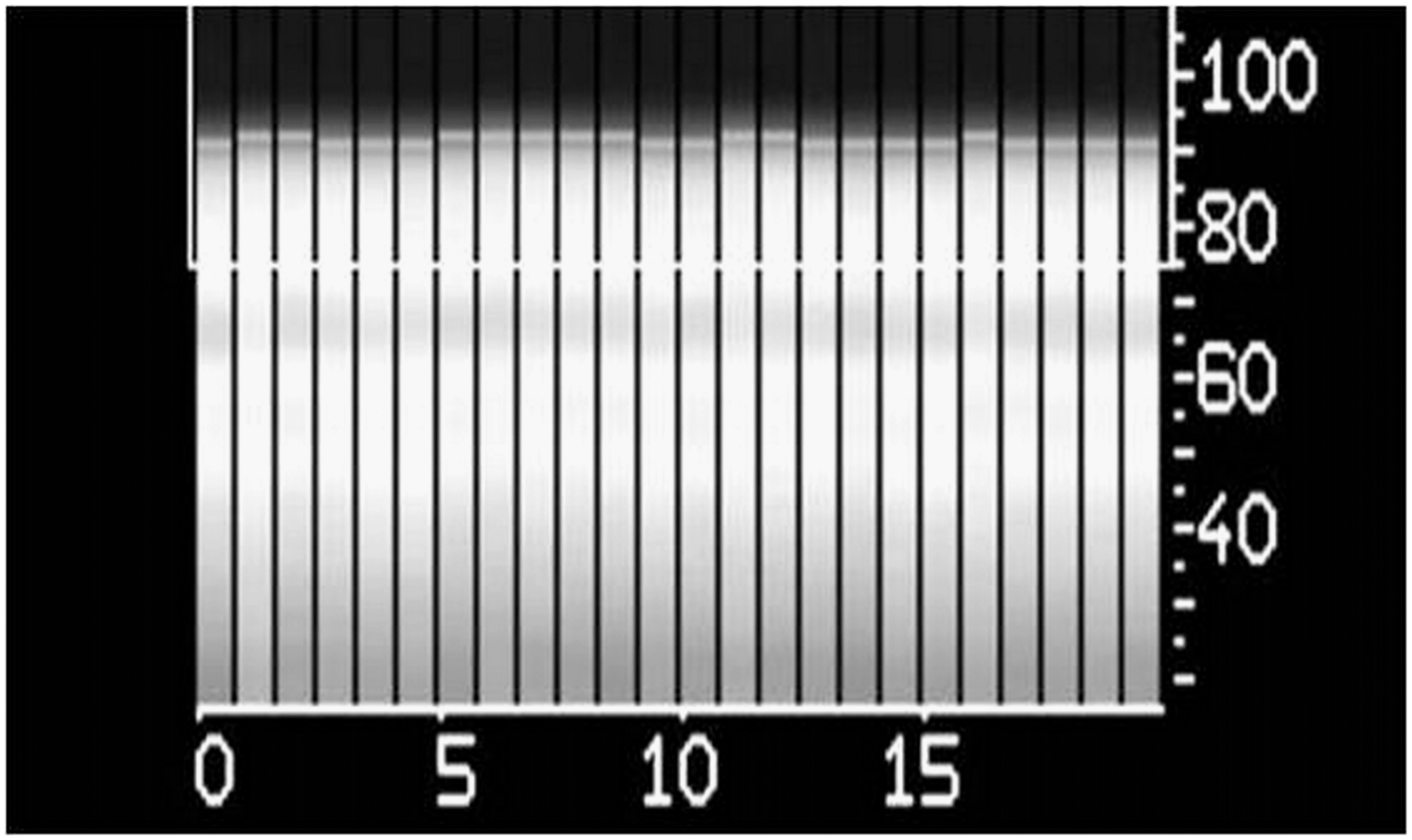
Navigator technique showing a projection of the liver dome position on the superior-inferior axis at each heart beat demonstrating the almost perfect stabilization of the chest during VP-MR, conversely to normal respiratory cycle. The x axis represents the time and the y axis the height in millimeters.

Participants underwent 3 preliminary training sessions with the technique before undergoing MRI with HFPV^®^. During MR acquisition, the stabilization period ended when the volunteer/patient decided to take a breath. Cardiac frequency and oxygen saturation were monitored throughout the test. The breathing cycle amplitude reduction was monitored by using navigator technique (Fig 2). Procedure tolerance was evaluated by the volunteers using an analog scale with the following values: 1 = inacceptable, 2 = bad, 3 = good, 4 = very good, 5 = excellent. In addition, the delineation of the lung-liver interface was quantified in the ultra-short echo time images by measuring the normalized maximum of the first derivative (MD) [11]. This measure is defined such that values greater than 1 indicate an improvement in image sharpness under prolonged thoracic stabilization relative to free breathing acquisition.

## Results

### VP-MR duration and tolerance for the 2 participants

The time between the start of procedure to the stabilization phase was 8 min and 6.5 min, respectively for volunteer 1 and patient 1. Three consecutive stabilization phases of 12, 11 and 8.5 min were registered for Volunteer 1. Four consecutive stabilization phases of 5, 6.2, 5.8 and 5.8 min were registered for Patient 1. Tolerance of the VP-MR examination was counted as 3 (good) for both participants.

### VP-MR images

**Fig 3:**
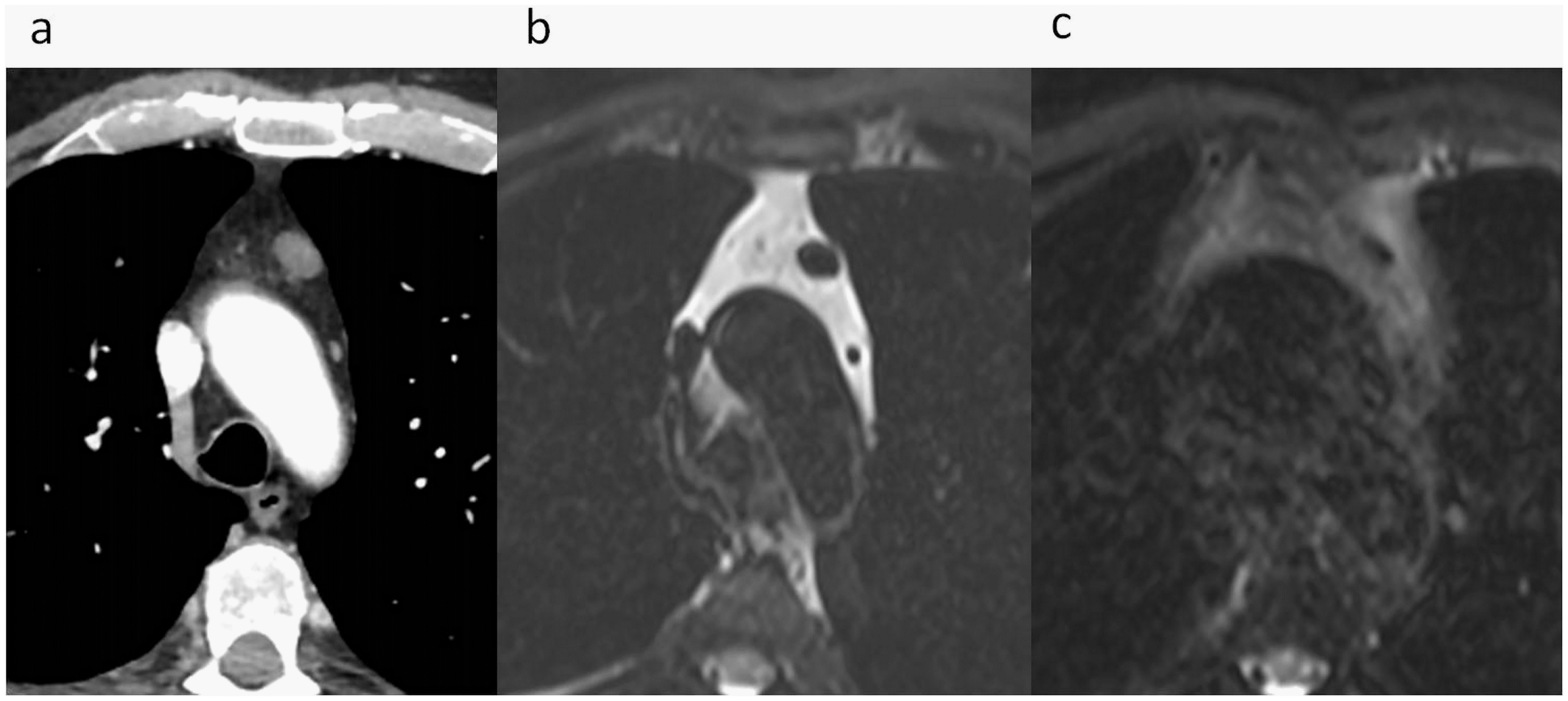
CT scan with iodine administration showing a nodule in the anterior mediastinum suspected being of thymic nature. (a). Fast spin echo T2 sequence at the same level with VP (b) and without (c). The thymic nodule is much better delineated under VP in b) than in c).

**Fig 4:**
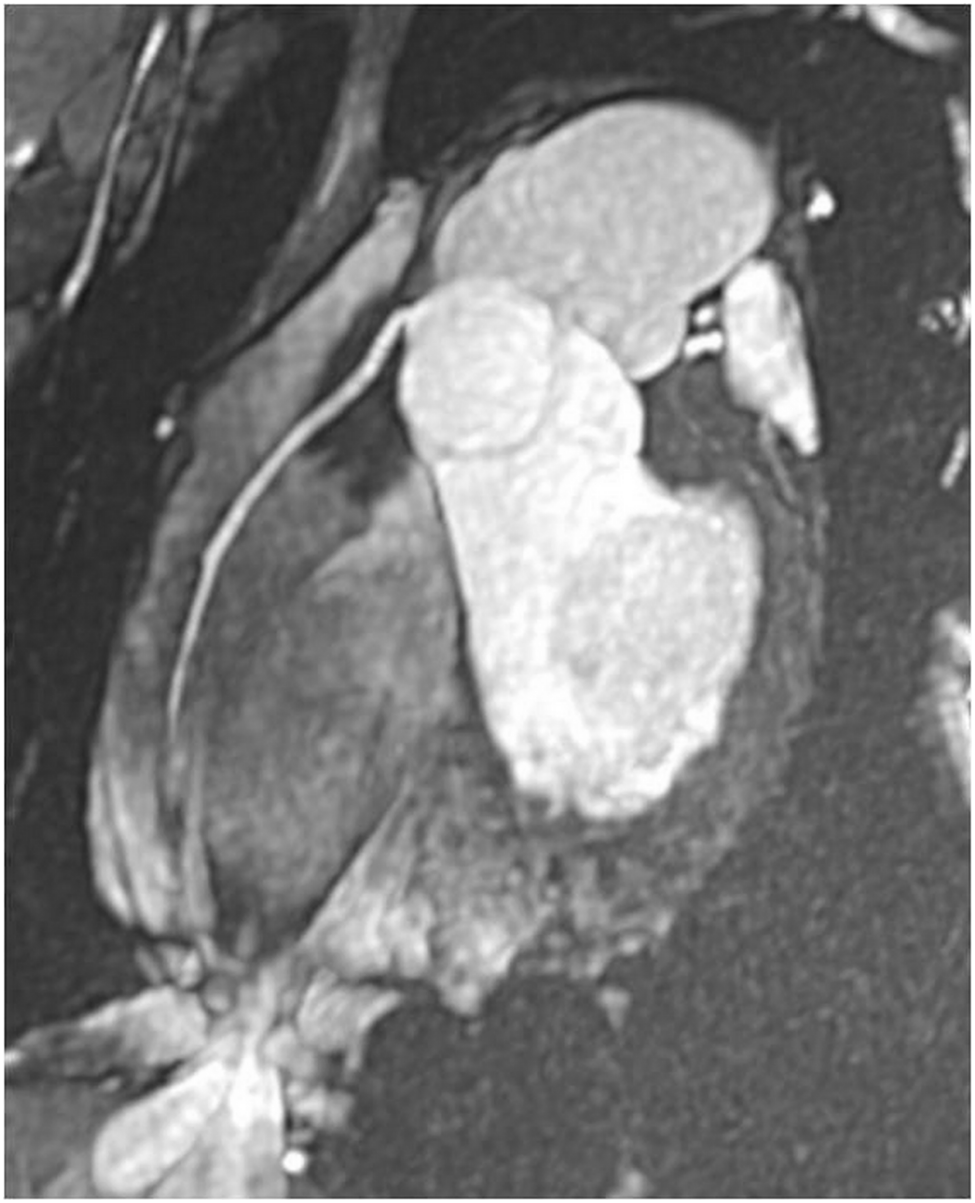
TRUFI 3D 1.3mm thick-slices in double obliquity focused on the heart. 15 mm thick MIP reformat perfectly showing the right coronary artery without blurring artefacts—due to thoracic stabilization associated with decreased diaphragmatic motion.

**Fig 5:**
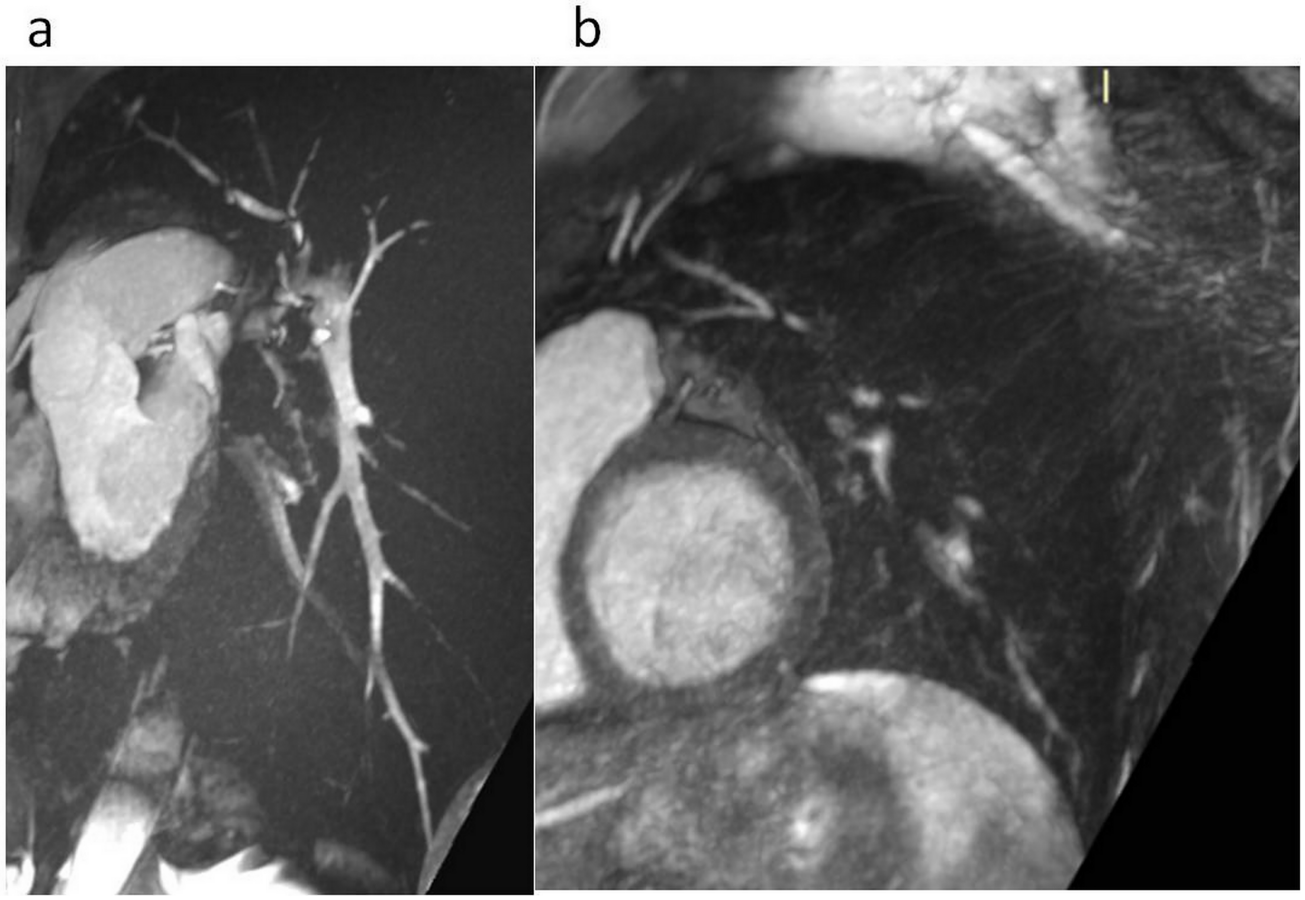
3D radial sequence in a sagittal oblique 20 mm thick MIP reformat showing the pulmonary vessels of the left lower lobe until their distality with VP. (a). Conversely, this couldn’t be obtained without VP (b). Note the quality of the endoluminal signal although no contrast was administrated.

**Fig 6:**
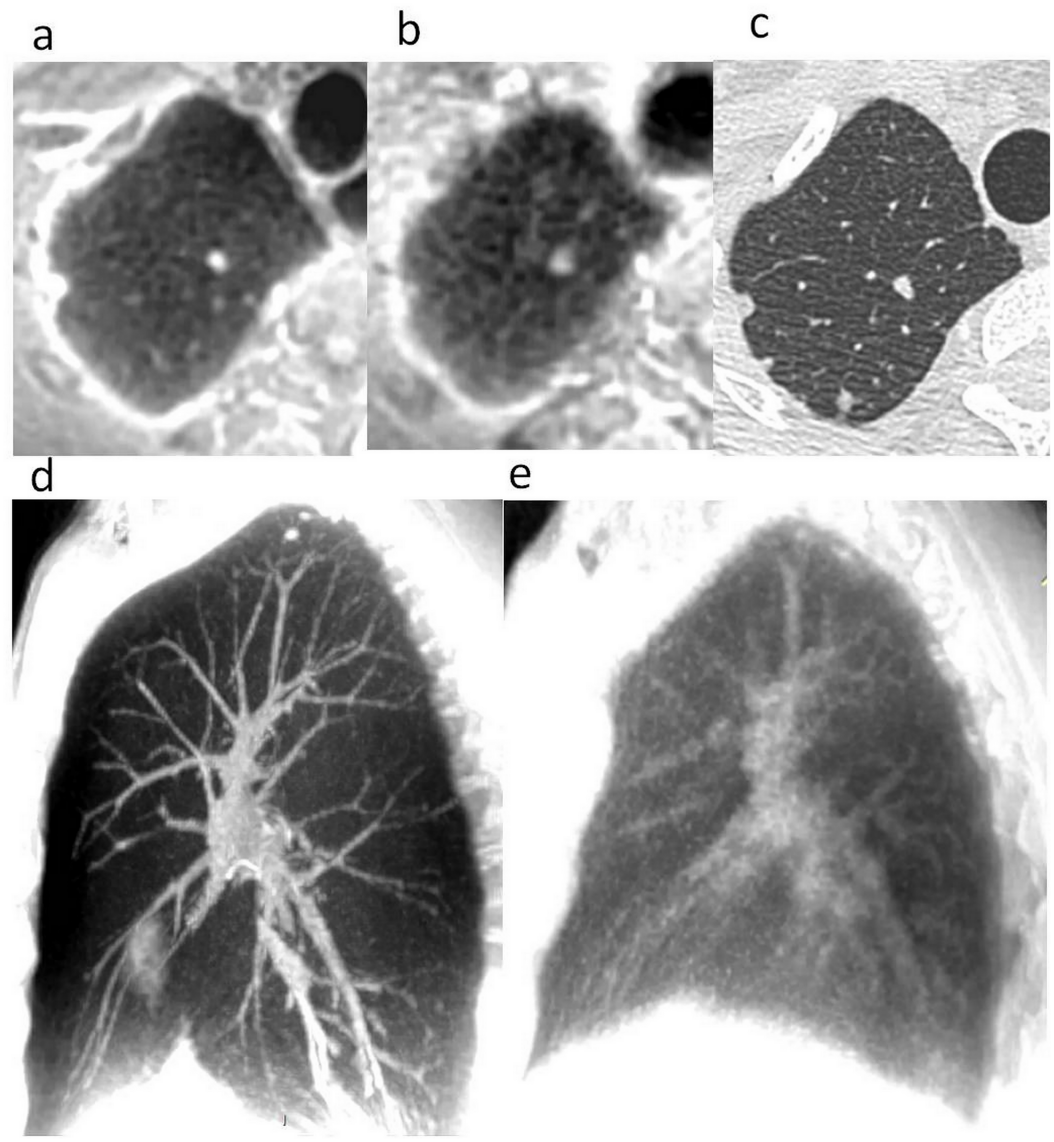
Ultra short time echo sequence at the level of the apex of the lung with VP. (a) and without VP (b). Incidental discovery of apical lung nodules that are more easily assessed in a) with sharper borders. Note that the lung parenchyma appears noisier in b). Correlation with thin CT slice in lung windowing (c) demonstrating the higher spatial resolution of CT with some additional details not detected by either MR sequence. Ultra short time echo sequence in sagittal 35 mm thick MIP reformat focused on the right lung. Vessels were much better assessed with increased sharpness of interfaces with VP (d) than without (e).

Image quality of all sequences was markedly improved under VP-MR. This concerned the mediastinum (Fig 3) as well as the coronary arteries (Fig 4), the pulmonary vessels (Fig 5), the bronchi, the lung parenchyma (Fig 6) and the chest wall. The quality of inspiration was linked to almost complete suppression of breathing artefacts while combined with an elongated aspect of the whole chest, in particular the heart and vessels (Figs 5 and 6). The T2 Fast spin echo sequence, which normally cannot be used in routine practice given its respiratory artefacts sensitivity, allowed to perfectly delineate the thymic lesion in Patient 1 (Fig 3). Some artefacts related to the VP technique were observed near the diaphragm. While the abnormal known mediastinal mass was perfectly assessed for Patient 1, incidental nodules were discovered in Volunteer 1 and confirmed with CT (Fig 6). Lung-liver interface delineation was improved in both subjects with MD values of 2.4±0.4 measured in Volunteer 1 and 1.6±0.4 in Patient 1.

## Discussion

The great sensitivity of chest MRI to respiratory motion has always been a technical challenge owing to long duration sequences required by low proton density, and high magnetic susceptibility of the lungs.

Respiratory triggering or gating techniques, such as navigator gating, can be used to suppress breathing artefacts, but at the price of increased total scan duration with acquisitions performed at end expiration, limiting the ability to analyze the lung. Breath holding is more efficient but intrinsically limits the duration of the acquisition. Multiple breath holds may be used for sequences requiring long acquisition duration. This however leads to increased total examination time with potential misregistration artefacts related to imperfect reproducibility of the breath hold volume thus impairing diagnostic quality of the images. While parallel imaging techniques can be used to shorten acquisition time, they also result in a decrease in signal to noise ratio (S/N). With Lung MRI using an MR-Compatible Active Breathing Control (MR-ABC), total imaging time could be reduced compared to navigator gating or respiratory belt use. ABC has been used to ensure reproducibility of breath hold volumes [12] but still requires segmenting of the examination in multiple steps. Our method presents the advantage of a long stability period during which multiple datasets covering the entire chest may be acquired at full inspiration.

Each thoracic stabilization had a respective average duration of about 10 min for Volunteer 1 and 5 to 6 min for Patient 1. To our knowledge, such a duration of thoracic stabilization has never been used in chest MR imaging. This could potentially shorten the total acquisition time to achieve whole-chest coverage while reducing the efforts of the patients and avoiding misregistration artefacts [13]. In addition, almost freezing the lung volume in full inspiration allowed the avoidance of blurring and ghosting artefacts by overall reducing the motion within voxels. In this way, an improvement of the S/N ratio with the use of signal averaging could be further expected by extending the time of a specific sequence. The potential to strictly compare various contrasts according to the sequences performed during the same stabilization period could also allow accurately merging/ the various signals with integration of diffusion parameters that could potentially improve diagnostic proposals and assessment of evolution under treatment.

As the acquisitions were performed at full inspiration, chest components were assessed in conditions similar to those obtained in routine chest CT. The motion suppression with improvement in spatial resolution resulted in a better delineation of all chest components.

Although some sequences such as conventional spin echo T2 have demonstrated their usefulness in the characterization of masses as cystic or solid, their use is unconceivable with classic chest MRI due to the couple of minutes acquisition time required [2]. Owing to the duration of the stabilization period approaching apnea with this new technique, the use of a T2 FSE allowed us to perfectly delineate the thymic lesion while using VP-MR technique compared with the sequence without VP technique in patient 1, which was similar to the CT appearance (Fig 3).

Another potential interest of VP-MR in full inspiration was the high signal within the blood pool obtained without contrast using the 3D radial Steady-State Free Precession (SSFP) acquisition that allowed displaying straight pulmonary vessels until distality, as well as the coronary arteries. The interest of 3D-SSFP sequence with respiratory self-navigation for whole-heart bright-blood coronary MRI with automatic segmentation of the blood pool with a slice thickness of 1.15 mm and isotropic voxels has already been demonstrated for the exploration of coronary arteries [14]. However, when applied to pulmonary vessels, the sinuosity of vessels reflecting the acquisition at a tidal volume currently precludes a complete assessment of disorders such as pulmonary emboli or pulmonary hypertension.

Regarding lung analysis, although new sequences have recently been developed [13,15], spatial resolution remains still a challenge, with a rate of detection of nodules between 35 and 96% [12,15]. The potential of acquisition at full inspiration would help to avoid their potential non recognition on expiration. Nodules detected in Volunteer 1 were confirmed by a low-dose CT (Fig 5). In the study of Heye et al. [13], despite the promising results of the new parallel imaging ultrafast SSFP sequence allowing for imaging in one breath-hold of 18–20 seconds with 1.9 mm isotropic spatial resolution, there were numerous false-positive nodules related to slightly blurred pulmonary vessels, possibly caused by motion affecting the MR signal. According to the authors, MR with long apnoea at full inspiration could overcome these limits [13].

Diffusion sequences didn’t show any abnormal signal within the detected nodules in Volunteer 1, precluding further evaluation. Such sequences, known to be penalized by motion and/or respiratory artefacts in uncooperative or dyspneic patients [16,17], could benefit from VP-MR.

An obvious limitation of our study is the low number of participants. However, this is aligned with the goal of this pilot experience which was to perform a first evaluation of HFPV^®^ with MRI. These preliminary data suggest relatively good reproducibility of the technique which is in agreement with the recent data obtained for chest radiotherapy [10]. Nevertheless, it must be emphasized that further evaluation is required to explore how VP-MR technique could perform in a large sample size with various pulmonary disorders. In fact, the effectiveness and tolerance of the HFPV^®^ has to be confirmed in subjects with lung disease, having a potential for overdistension in obstructive and underventilation in restrictive diseases. In addition, the acquisition still requires significant training of the subject and this technique might not be tolerated by some patients, particularly if applied in the MRI environment as described in this pilot study. However, as published by Peguret et al. [10], which is the first report of this technique outside the MRI, this high frequency percussion ventilation system could be successfully applied in nearly all the tested cases. Furthermore, as no contrast agent was used, the potential of the technique after gadolinium injection has not been evaluated. Similarly, perfusion and ventilation were not studied.

## Conclusion

In this pilot experience, Pulsatile Flow Ventilation^™^ was successfully applied to induce prolonged stabilization of lung volumes with marked reduction of respiratory motion during MRI acquisitions at full inspiration. This resulted in the suppression of most respiratory-related artefacts while allowing the exploration of the whole chest at full inspiration phase with increased spatial resolution. These promising results open the field for new applications of thoracic MRI, in particular the follow-up of interstitial lung disease, bronchial or infectious disorders as well as the assessment of mediastinal masses, pulmonary artery or coronary artery disorders. These results call for developing studies to establish the real clinical value of VP-MR while improving the technique.

## Supporting information

S1 Images DICOM data from Volunteer 1 (1st half). (ZIP)

S2 Images DICOM data from Volunteer 1 (2nd half). (ZIP)

S3 Images DICOM data from Patient 1 (1st half). (ZIP)

S4 Images DICOM data from Patient 1 (2nd half). (ZIP)

S1 Table Used for the quantification of lung/liver interfaces. (PDF)
