# Supplementary material for: Chest-MRI under pulsatile flow ventilation: A new promising technique
Source: PLoS One. 2017 Jun 12;12(6):e0178807. doi: 10.1371/journal.pone.0178807 (PMC5467845; doi:10.1371/journal.pone.0178807)
Supplement: S1 Table — (PDF) [file pone.0178807.s005.pdf]

Volunteer 1			
Profile #	Max derivative (UTE Free breathing)	Max derivative (UTE VP-MR)	Normalization (VP-MR/Free Breating
1	7.5	15	2.0
2	8.5	16	1.9
3	8.5	16.5	1.9
4	6.5	17.5	2.7
5	7	19	2.7
6	6.5	19	2.9
7	9.5	19	2.0
8	8	18.5	2.3
9	6	19	3.2
10	7	18	2.6
11	7	16.5	2.4
12	6.5	17.5	2.7
13	7	16.5	2.4
14	7.5	15.5	2.1
15	8	17	2.1
16	6.5	19.5	3.0
17	9	18.5	2.1
18	9	19	2.1
19	10	22.5	2.3
20	7	22	3.1
21	8.5	16	1.9
		Average	2.4
		Standard deviation	0.4

Patient 1			
Profile #	Max derivative (UTE Free breathing)	Max derivative (UTE VP-MR)	Normalization (VP-MR/Free Breating
1	8	10	1.3
2	7.5	10	1.3
3	8.5	10	1.2
4	9	10	1.1
5	6.5	12.5	1.9
6	7.5	12.5	1.7
7	7.5	14	1.9
8	9	13	1.4
9	9.5	10	1.1
10	8.5	13.5	1.6
11	8	15	1.9
12	12.5	14.5	1.2
13	8	14.5	1.8
14	6.5	13	2.0
15	6.5	14	2.2
16	8	15.5	1.9
17	6.5	17.5	2.7
18	9	14.5	1.6
19	9.5	13	1.4
20	8	13	1.6
21	8	13	1.6
		Average	1.6
		Standard deviation	0.4
